# Associations Between Diet Quality and Proinflammatory Cytokines in Newly Diagnosed Head and Neck Cancer Survivors

**DOI:** 10.1016/j.cdnut.2023.102015

**Published:** 2023-10-12

**Authors:** Christian A. Maino Vieytes, Laura S. Rozek, Gregory T. Wolf, Anna E. Arthur

**Affiliations:** 1 Division of Nutritional Sciences, University of Illinois at Urbana-Champaign, Urbana, IL, United States; 2 Lombardi Comprehensive Cancer Center, Georgetown University, Washington, DC, United States; 3 Department of Otolaryngology, University of Michigan, Ann Arbor, MI, United States; 4 Department of Dietetics and Nutrition, University of Kansas Medical Center, Kansas City, KS, United States

**Keywords:** survivorship, cancer, nutritional epidemiology, biomarkers, inflammation, cytokines, dietary quality indices

## Abstract

**Background:**

Head and neck squamous cell carcinoma (HNSCC) is a class of heterogenous cancers involving the upper aerodigestive tract. We previously demonstrated the utility of *a priori* diet quality indices for predicting survival after an HNSCC diagnosis. The aim of this analysis was to evaluate the role of those *a priori* diet quality indices and proinflammatory cytokines in newly diagnosed HNSCC survivors.

**Methods:**

We analyzed cross-sectional data from a sample (*n* = 146; mean age 59.6 y; 79.3% male) from the University of Michigan Head and Neck Specialized Program of Research Excellence prospective longitudinal cohort study. Dietary intake was measured at pretreatment using a food frequency questionnaire. Serum samples were also collected at pretreatment. Covariate-adjusted proportional odds and logistic regression models were used to assess the relationship between 6 diet quality indices (Alternative Healthy Eating Index [AHEI]-2010, Alternate Mediterranean Diet, Dietary Approaches to Stop Hypertension [DASH], and 3 low-carbohydrate indices) and serum measures of a panel of 10 inflammatory cytokines and a cytokine summary composite score.

**Results:**

Higher scores on the AHEI-2010 and DASH diet quality indices were associated with higher odds of lower cytokine value scores for several cytokines and for the cytokine summary composite score (AHEI-2010—odds ratio [OR]: 1.55; 95% confidence interval [CI]: 1.10, 2.20; DASH—OR: 1.65; 95% CI 1.15, 2.36).

**Conclusions:**

Higher scores on the AHEI-2010 and DASH diet quality indices may be associated with lower proinflammatory cytokine levels in HNSCC survivors.

## Introduction

Proinflammatory cytokines are proteins normally translated and secreted by immune cells in response to stressful stimuli, such as infection, implicated in cancer initiation, progression, and metastasis [1]. The tumor microenvironment promotes the secretion of several cytokines that fulfill paracrine and autocrine roles. Constitutive production of these proinflammatory cytokines results in a state of chronic inflammation, whereby those cytokines are persistently detected in the systemic circulation and are a classic phenotype of cancer [1]. Within the context of head and neck squamous cell carcinoma (HNSCC), proinflammatory cytokines including the IL-1,4, 6, and 8, hepatocyte growth factor (HGF), interferon-γ (IFN-γ), and vascular endothelial growth factor (VEGF) have all been implicated in tumor-survival tactics including immune evasion, angiogenesis, chemoattraction, and metastasis [[2], [3], [4]]. Notably, elevation in many of these cytokines has been implicated in less favorable outcomes, such as a diminished response to therapy and survival [[5], [6], [7], [8]]. Consequently, several proinflammatory cytokines have attracted attention as potential targets for cancer therapeutics [9].

The regulation of proinflammatory cytokine production is governed typically at the transcriptional level. Thus, regulation involves an interplay of major cellular pathways comprised of a bounty of transcription factors involved in growth, such as nuclear factor kappa-light-chain-enhancer of activated B cells (NF-κB). For instance, the IL-6 promoter region contains an NF-κB binding site [10]. Therapeutics have focused on targeting these pathways upstream of cytokine production. However, this evidence has also generated interest in dietary components understood to influence pathway transduction that function as chemopreventive agents.

Diets comprising various fruits and vegetables have been associated with bolstered prognosis in previous studies of HNSCC survivors, defined as individuals diagnosed with cancer from the time of diagnosis through the balance of life [[11], [12], [13], [14], [15]]. Many *a priori* diet quality indices used in the nutritional epidemiology literature emphasize the consumption of fruits and vegetables, although there is otherwise significant nuance in their computation. In previous analyses, we demonstrated the utility of select *a priori* diet quality indices for predicting cancer-related outcomes, including survival and symptomatology, in a cohort of newly diagnosed HNSCC patients [16,17]. We also showed that dietary patterns derived using *a posteriori* methods, i.e., employing principal components analysis to empirically extract dietary patterns from observed food frequency questionnaire (FFQ) data, and serum micronutrient concentrations were associated with serum proinflammatory cytokine levels in newly diagnosed HNSCC survivors [18]. Nevertheless, the question of whether some *a priori* diet quality indices, i.e., indices that measure adherence to a prespecified set of guidelines or dietary patterns, are associated with more favorable profiles of serum proinflammatory cytokines has not been broached in the HNSCC study population.

Given the practical nature of *a priori* diet quality indices and the ability to easily translate them into dietary recommendations, we investigated the influence of 6 indices on serum inflammatory cytokines in newly diagnosed HNSCC patients [19]. This analysis follows our previous work that demonstrated a significant survival advantage for those HNSCC subjects with higher scores on the Alternative Healthy Eating Index (AHEI)-2010 [16]. Considering these previous findings, we hypothesized that the pretreatment adherence to, i.e., a higher score on, the AHEI-2010 would be inversely associated with pretreatment proinflammatory cytokine levels and that we would not see any significant associations involving the remaining indices in this sample of newly diagnosed HNSCC survivors.

## Methods

### Study design and population

This analysis used cross-sectional patient data from the University of Michigan Head and Neck Specialized Program of Research Excellence (UM-SPORE). The UM-SPORE is a longitudinal cohort study (Supplementary Figure 5) that recruited newly diagnosed and previously untreated HNSCC patients through the University of Michigan Hospital System between 2008 and 2014 and aimed to identify prognostic factors and garner greater knowledge of the disease process of HNSCC. Subjects were screened and excluded from the study if: *1*) their age was less than 18 y; *2*) they were pregnant; *3*) they were a non-English speaker; *4*) they had a previously diagnosed mental disorder; *5*) they had a previous or concomitant diagnosis of a tumor in the non-upper aerodigestive tract; and *6*) they had another primary HNSCC diagnosed within the last 5 y. All study procedures complied with the standards outlined by the University of Michigan Institutional Review Board (approval number for which consent was granted for obtaining and analyzing the data is HUM00042189) and the Helsinki Declaration of 1975. All study participants provided written consent to participate in the study.

There were 209 consenting subjects who completed an FFQ and provided a venous blood draw. We also removed subjects with estimated caloric intakes of <200 kcal and >5000 kcal, given the implausibility of such intakes (*n* = 49) and those with tumors in anatomical sites other than the oropharynx, larynx, hypopharynx, or oral cavity (*n* = 14) [20]. The final analytical sample included 146 newly diagnosed HNSCC survivors.

### Explanatory variables: diet quality index scores

All analyses used pretreatment data from the UM-SPORE study. Dietary intake data were collected at study entry but prior to the initiation of treatment, i.e., pretreatment, using the self-administered 2007 semiquantitative Harvard FFQ. This 131-item FFQ is designed to capture the usual intake of several commonly consumed foods over the previous year of the participant’s life [21,22].

Six *a priori* diet quality indices were chosen for the analysis. These included the AHEI-2010, the Alternate Mediterranean Index (aMED), the Dietary Approaches to Stop Hypertension (DASH), a low-carbohydrate index, a low-carbohydrate index that emphasized animal sources of fat and protein, and a low-carbohydrate index that emphasized plant sources of fat and protein [[23], [24], [25]]. We previously described these indices and their computational algorithms but have included Supplementary Figures 1–4 describing the food components involved in the calculations [16]. These indices were computed using estimated daily servings data compiled from the pretreatment 131-item 2007 Harvard FFQ that participants completed at study entry. Energy intake was estimated by accounting for a food item’s nutrient content, the portion size described in the FFQ, and the subject’s selected frequency of intake. All diet quality scores were adjusted for total energy intake before any analyses using the nutrient residual model described by Willet et al. [26]. We note that 41 participants lacked the complete data to compute the plant-based low-carbohydrate index score. Therefore, analyses of this diet quality index involved 105 subjects only. The results are appropriately annotated to make this clear.

### Covariates

Upon study enrollment, subjects completed a self-administered health questionnaire that probed demographic and lifestyle factors such as smoking history, drinking history, weight status, sleep quality, and other comorbid conditions. Clinical tumor stage and site data were obtained through an electronic medical record review. Sociodemographic characteristics included in our analysis were sex and age. Clinical covariates included BMI (kg/m^2^), tumor stage (Stages 0, I, II compared with Stages III and IV), tumor site (larynx/hypopharynx, oral cavity, or oropharynx), human papillomavirus (HPV) status, and the Adult Comorbidity Evaluation (ACE)-27 score. The behavioral and lifestyle characteristics of interest were smoking and drinking status (both categorized as current, former, or never). Selection of covariates to include in these models was done *a priori*.

### Dependent variables: serum cytokines

Venous blood samples were collected from a subset of participants using conventional venipuncture procedures (30 mL). These samples were centrifuged, and sera were isolated, retained in 0.5-mL aliquots, and stored at −80 °C. Study personnel were blinded to the serum samples by assigning all samples a bar code prior to storage in the UM-SPORE Tissue Core.

A panel of proinflammatory cytokines was assayed on patient serum samples using paired-antibody enzyme-linked immunosorbent kits. All samples were analyzed in the same batch, and internal controls were used in all assay plates. The procedure proceeded as follows: patient serum aliquots stored at −80°C were thawed and incubated overnight at 4°C on microtiter plates that contained monoclonal antibodies targeted for IL-6, IL-8, IL-10, IL-17, growth-related oncogene (GRO)-β, HGF, IFN-γ, transforming growth factor-β, TNF-α, and VEGF. Unbound residues were rinsed off, and cytokine-specific polyclonal antibodies were added. The plates were incubated at room temperature for 2 h, after which they were rinsed and incubated again for another hour in streptavidin horseradish peroxidase. Finally, a substrate solution was introduced, and color development was allowed to progress for 25 min. We used a microplate reader to evaluate the colorimetric density of the samples while comparing them to a standard curve. As a further measure of quality control, we prepared multiple samples from each subject, including one sample per subject per batch.

In addition to analyzing each cytokine individually as a response variable, we computed a summary composite score comprised of cytokines in the panel. Specifically, we calculated Z scores for each participant for each cytokine, as previously done by colleagues [27]. This marker of overall inflammation was computed as follows using only proinflammatory cytokines:

(1) CytokineSummaryScore=Z(IL6)+Z(IL8)+Z(IL17)+Z(GROβ)+Z(HGF)+Z(IFNγ)+Z(TNFα)+Z(VEGF)

### Statistical analysis

Descriptive statistics for demographic, clinical, and behavioral characteristics were tabulated. Additionally, we evaluated the distribution of those characteristics across high and low fractions of the diet quality indices after a median split. For the primary analysis, we used a combination of methods involving generalized linear models that were dependent on the outcome variable (cytokine) being modeled. We binned cytokines with a percentage of values equaling zero being greater than 50% (TNF-α and IL-17) into a set of binary categories (zero and nonzero) and used binary logistic regression to model the log-odds of having a nonzero count of those cytokines as a function of the diet quality indices alongside relevant confounders. For all other cytokines, we binned observations into 3 categories based on tertile cutoff points and fit proportional-odds models (using the proportional-odds assumption) for these ordinal factors. We implemented this scheme of outcome categorization to bolster the reproducibility of the analysis and with careful attention to the distributions of each outcome variable. We used several model specifications within the general model forms below, where Equation 2 shows the model form for the binary logistic regression models, and Equation 3 shows the model form for the proportional-odds models:

(2) $$logit\left[\mathrm{Pr}\;{\left(Y=1\;|\;x\right)}_{i}\right]=\alpha +\sum_{k=1}^{t}\beta_{k}x_{ki}+\sum_{k=t+1}^{p}\beta_{k}x_{ki}$$

$$logit\left[\mathrm{Pr}\;{\left(Y\leq j\;|\;x\right)}_{i}\right]=\alpha_{j}+\sum_{k=1}^{t}\beta_{k}x_{ki}+\sum_{k=t+1}^{p}\beta_{k}x_{ki}$$

(3) wherej∈{1…J−1}andJ=3

where t varied between models, the subscript k referred to dummy-coded variables for the levels of the primary explanatory variables (diet quality index quantiles) or single continuous variables up to t, where k also corresponded to a covariate or a dummy-coded variable for a level of a covariate factor between k=t+1 to p, and p denoted the total number of variables in the model. First, we modeled the dietary patterns using categorical specifications (quartiles, t = 3, Equations 2 and 3). We conducted a test for linear trend across the quartiles of the dietary patterns by assigning subjects the median of their respective quartile and then modeling as a continuous variable (t = 1, Equations 2 and 3). Second, we specified the dietary pattern raw scores scaled by their standard deviations and modeled them as continuous variables (t = 1, Equations 2 and 3). Third, we used a basis expansion on the dietary covariate for natural cubic splines with 4 interior knots. We assessed for nonlinearity using the likelihood ratio test—comparing the model with the scaled dietary covariate and the model with spline terms [28,29]. All models were adjusted for age, sex, BMI, HPV status, tumor site, stage, estimated caloric intake, smoking status, and drinking status. We did not adjust for race and ethnicity given that the sample was nearly homogenous (non-Hispanic White) and decisions on covariates to include in the models were *a priori*. Education status was correlated with other covariates (i.e., smoking, drinking, and tumor site) and was thus not included. We considered several other covariates listed above, but a decision was made to exclude them given the relatively small sample size and concerns for statistical power. Nevertheless, the set of covariates we adjusted for included the most relevant covariates used in studies of HNSCC survivors. The R code for reproducing the analyses is publicly available and found in a GitHub repository [30].

## Results

### Descriptive analysis

Table 1 documents the epidemiological characteristics of the study sample. The analytic sample comprised a majority of male subjects and non-Hispanic White respondents. Clinically, most subjects were in advanced stages (Stages III–IV) of their cancer, and most cases had an HPV-negative status. Concerning behavioral characteristics, most subjects reported being current or former smokers and drinkers.

**TABLE 1:** Epidemiologic characteristics of the study sample at pretreatment (*n* = 146)

Characteristic	Frequency (%) or Mean (SD)
Mean age, y	59.6 (11.2)
Sex^1^	
Male	115 (79.3)
Race and ethnicity^2^	
African American	3 (2.1)
American Indian/Eskimo/Aleutian	1 (0.7)
Hispanic/Latinx	4 (2.8)
Non-Hispanic White	136 (93.8)
Other	1 (0.7)
Mean body mass index (kg/m^2^)	27 (5.5)
Tumor site	
Larynx	38 (26.0)
Oral cavity	50 (34.2)
Oropharynx	58 (39.7)
Tumor stage	
Stages 0–II	26 (17.8)
Stage III–IV	120 (82.2)
HPV status	
Negative	72 (49.3)
Positive	47 (32.2)
Equivocal	27 (18.5)
ACE score	
Moderate/severe	44 (30.1)
None/mild	102 (69.9)
Smoking status	
Current	61 (41.8)
Former	53 (36.3)
Never	32 (21.9)
Drinking status^3^	
Current	102 (69.9)
Former	36 (24.7)
Never	8 (5.5)
Mean AHEI Score	58.5 (11.5)
Mean aMED Index Score	4.1 (2.1)
Mean DASH Index Score	23.9 (4.7)
Mean Low-Carbohydrate Index Score	14.9 (7.2)
Mean Animal-Based Low Carbohydrate Index Score	15 (7.6)
Mean Plant-Based Low Carbohydrate Index Score	14.9 (6.0)

ACE, Adult Comorbidity Evaluation; AHEI, Alternative Healthy Eating Index; aMED, Alternate Mediterranean Diet; DASH, Dietary Approaches to Stop Hypertension; HPV, human papillomavirus.

Percentages may not add to 100% given rounding

1 *n* = 1 missing values

2 *n* = 3 missing values

3 Current drinker is defined as any alcohol consumption in the past year

In Table 2, we detail the distributions of the epidemiological characteristics across upper and lower fractions (split at the median) of the diet quality indices used in the study. A greater proportion of participants in the higher fractions of the AHEI-2010, aMED, and DASH indices had tumors in the oropharynx compared to the 3 low-carbohydrate indices. Participants in the lower fractions of all the diet quality indices examined except the plant-based low-carbohydrate index also had more advanced cancers (Stages III–IV). There were no appreciable differences in ACE-27 score across upper and lower fractions of the diet quality indices except for the animal-based low-carbohydrate index.

**TABLE 2:** Epidemiological characteristics of the study sample stratified on higher and lower fractions of the diet quality indices^1^

Characteristic	AHEI-2010		aMED		DASH		Low Carbohydrate (LC)		Animal-Based LC		Plant-Based LC^2^	
	M1 (*n* = 72)	M2 (*n* = 73)	M1 (*n* = 73)	M2 (*n* = 73)	M1 (*n* = 73)	M2 (*n* = 73)	M1 (*n* = 73)	M2 (*n* = 73)	M1 (*n* = 73)	M2 (*n* = 73)	M1 (*n* = 52)	M2 (*n* = 53)
Age	59.4 (12.1)	60 (10.2)	59.1 (12.1)	60.1 (10.2)	59.7 (11.7)	59.5 (10.7)	58.7 (10.6)	60.5 (11.7)	59.8 (10.7)	59.5 (11.7)	59.7 (12.0)	62.6 (9.2)
Sex^3^												
Female	11 (15.3)	19 (26.0)	15 (20.8)	15 (20.5)	11 (15.1)	19 (26.4)	14 (19.4)	16 (21.9)	16 (22.2)	14 (19.2)	9 (17.6)	11 (20.8)
Male	61 (84.7)	54 (74.0)	57 (79.2)	58 (79.5)	62 (84.9)	53 (73.6)	58 (80.6)	57 (78.1)	56 (77.8)	59 (80.8)	42 (82.4)	42 (79.2)
Race and ethnicity^4^												
African American	0 (0.0)	3 (4.1)	2 (2.8)	1 (1.4)	1 (1.4)	2 (2.8)	1 (1.4)	2 (2.7)	1 (1.4)	2 (2.7)	0 (0.0)	3 (5.7)
American Indian/Eskimo/Aleutian	0 (0.0)	1 (1.4)	0 (0.0)	1 (1.4)	1 (1.4)	0 (0.0)	0 (0.0)	1 (1.4)	0 (0.0)	1 (1.4)	0 (0.0)	0 (0)
Hispanic/Latinx	2 (2.8)	2 (2.7)	2 (2.8)	2 (2.7)	1 (1.4)	3 (4.2)	3 (4.2)	1 (1.4)	3 (4.2)	1 (1.4)	0 (0.0)	1 (1.9)
Non-Hispanic White	70 (97.2)	66 (90.4)	68 (94.4)	68 (93.2)	70 (95.9)	66 (91.7)	67 (93.1)	69 (94.5)	67 (93.1)	69 (94.5)	50 (98.0)	49 (92.5)
Other	0 (0.0)	1 (1.4)	0 (0.0)	1 (1.4)	0 (0.0)	1 (1.4)	1 (1.4)	0 (0.0)	1 (1.4)	0 (0.0)	1 (2.0)	0 (0)
Body mass index (kg/m^2^)	26.8 (5.7)	27.3 (5.4)	26.7 (5.3)	27.3 (5.8)	26.8 (6.5)	27.3 (4.4)	26.2 (4.4)	27.9 (6.4)	26.2 (4.3)	27.9 (6.5)	27 (4.3)	27 (5.9)
Tumor site												
Larynx	19 (26.4)	19 (26.0)	22 (30.1)	16 (21.9)	24 (32.9)	14 (19.2)	16 (21.9)	22 (30.1)	17 (23.3)	21 (28.8)	12 (23.1)	18 (34)
Oral cavity	32 (44.4)	18 (24.7)	32 (43.8)	18 (24.7)	30 (41.1)	20 (27.4)	24 (32.9)	26 (35.6)	23 (31.5)	27 (37.0)	18 (34.6)	15 (28.3)
Oropharynx	21 (29.2)	36 (49.3)	19 (26.0)	39 (53.4)	19 (26.0)	39 (53.4)	33 (45.2)	25 (34.2)	33 (45.2)	25 (34.2)	22 (42.3)	20 (37.7)
Tumor stage												
Stage 0–II	10 (13.9)	16 (21.9)	10 (13.7)	16 (21.9)	8 (11.0)	18 (24.7)	7 (9.6)	19 (26.0)	10 (13.7)	16 (21.9)	12 (23.1)	8 (15.1)
Stage III–IV	62 (86.1)	57 (78.1)	63 (86.3)	57 (78.1)	65 (89.0)	55 (75.3)	66 (90.4)	54 (74.0)	63 (86.3)	57 (78.1)	40 (76.9)	45 (84.9)
HPV status												
Negative	40 (55.6)	32 (43.8)	44 (60.3)	28 (38.4)	42 (57.5)	30 (41.1)	34 (46.6)	38 (52.1)	31 (42.5)	41 (56.2)	25 (48.1)	23 (43.4)
Positive	19 (26.4)	27 (37.0)	20 (27.4)	27 (37.0)	19 (26.0)	28 (38.4)	25 (34.2)	22 (30.1)	27 (37.0)	20 (27.4)	15 (28.8)	18 (34)
Equivocal	13 (18.1)	14 (19.2)	9 (12.3)	18 (24.7)	12 (16.4)	15 (20.5)	14 (19.2)	13 (17.8)	15 (20.5)	12 (16.4)	12 (23.1)	12 (22.6)
ACE score												
Moderate/severe	22 (30.6)	22 (30.1)	21 (28.8)	23 (31.5)	22 (30.1)	22 (30.1)	24 (32.9)	20 (27.4)	26 (35.6)	18 (24.7)	19 (36.5)	16 (30.2)
None/mild	50 (69.4)	51 (69.9)	52 (71.2)	50 (68.5)	51 (69.9)	51 (69.9)	49 (67.1)	53 (72.6)	47 (64.4)	55 (75.3)	33 (63.5)	37 (69.8)
Smoking status												
Current	36 (50.0)	25 (34.2)	35 (47.9)	26 (35.6)	40 (54.8)	21 (28.8)	29 (39.7)	32 (43.8)	26 (35.6)	35 (47.9)	20 (38.5)	23 (43.4)
Former	22 (30.6)	31 (42.5)	23 (31.5)	30 (41.1)	20 (27.4)	33 (45.2)	28 (38.4)	25 (34.2)	31 (42.5)	22 (30.1)	19 (36.5)	18 (34)
Never	14 (19.4)	17 (23.3)	15 (20.5)	17 (23.3)	13 (17.8)	19 (26.0)	16 (21.9)	16 (21.9)	16 (21.9)	16 (21.9)	13 (25.0)	12 (22.6)
Drinking status^5^												
Current	49 (68.1)	52 (71.2)	51 (69.9)	51 (69.9)	54 (74.0)	48 (65.8)	50 (68.5)	52 (71.2)	47 (64.4)	55 (75.3)	38 (73.1)	39 (73.6)
Former	20 (27.8)	16 (21.9)	18 (24.7)	18 (24.7)	16 (21.9)	20 (27.4)	18 (24.7)	18 (24.7)	21 (28.8)	15 (20.5)	12 (23.1)	12 (22.6)
Never	3 (4.2)	5 (6.8)	4 (5.5)	4 (5.5)	3 (4.1)	5 (6.8)	5 (6.8)	3 (4.1)	5 (6.8)	3 (4.1)	2 (3.8)	2 (3.8)

ACE, Adult Comorbidity Evaluation; AHEI, Alternative Healthy Eating Index; aMED, Alternate Mediterranean Diet; DASH, Dietary Approaches to Stop Hypertension; HPV, human papillomavirus.

1 Fractions were determined using a median split. Percentages may not add to 100% given rounding.

2 *n* = 105 subjects with the computed index given that 41 subjects lacked the complete necessary data for computation of the full index.

3 *n* = 1 missing values

4 *n* = 3 missing values

5 Current drinker is defined as any alcohol consumption in the past year

### Cytokine analysis

**FIGURE 1:**
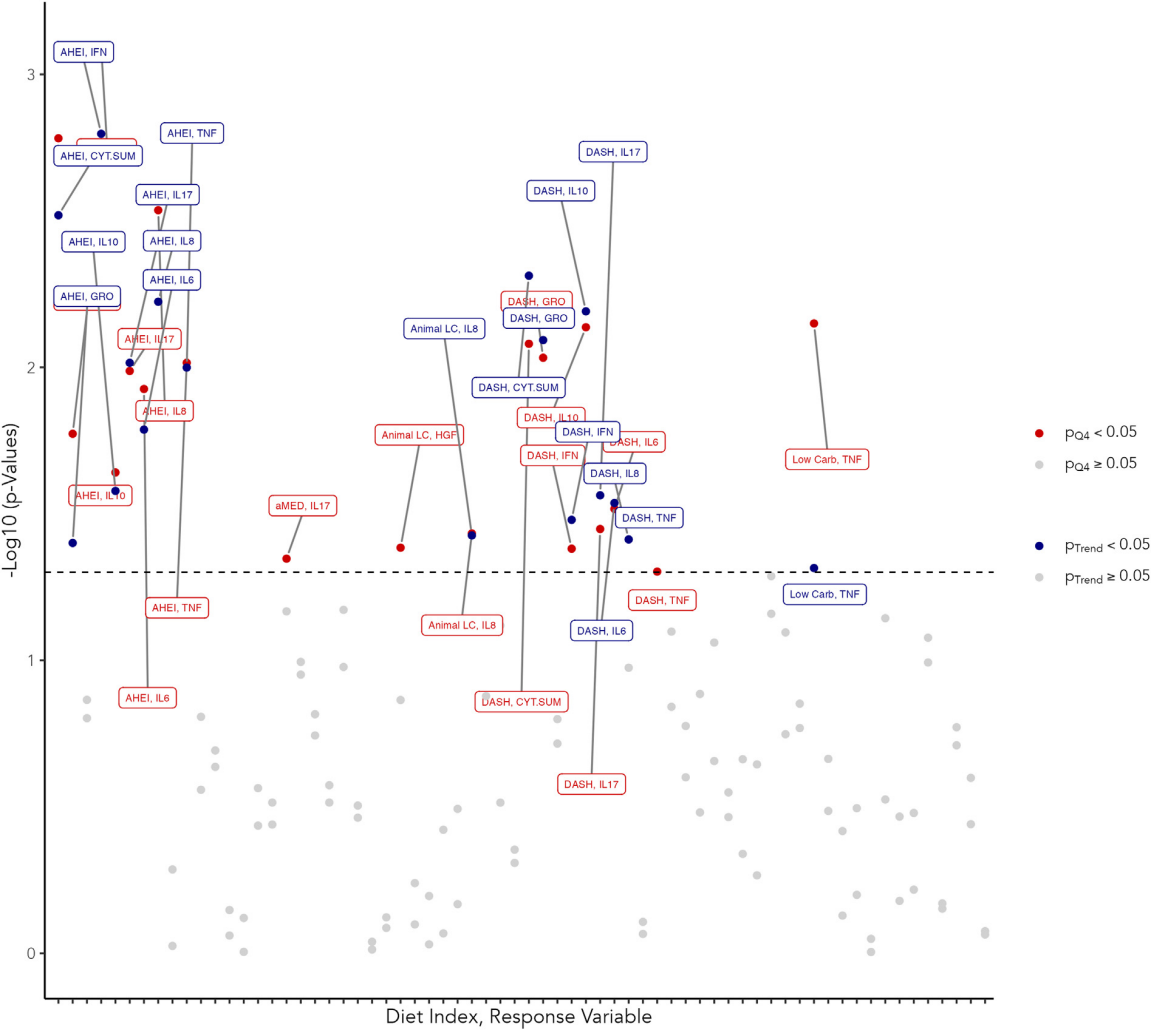
Labeled and log-scaled *P* values for the dummy-coded variables indicating the highest quartile of the diet indices and the trend variables in the tests for trend. We implemented proportional-odds models for evaluating the relationships between diet quality indices and 3-level factors of the proinflammatory cytokines. The exception was that binary logistic regression models were used for modeling TNF-α and IL-17 given the superfluous zero values. All models were adjusted for age, sex, BMI, tumor site, stage, HPV status, estimated caloric intake, smoking status, and drinking status. A dashed line indicates the threshold for α = 0.05. Abbreviations: AHEI, Alternative Healthy Eating Index; aMED, Alternate Mediterranean Diet; CYT.SUM, cytokine summary score; DASH, Dietary Approaches to Stop Hypertension; GRO, growth-related oncogene; HGF, hepatocyte growth factor; HPV, human papillomavirus; IFN, interferon; LC, low carbohydrate.

Several diet quality indices were significantly associated with the inflammatory indices examined. Figure 1 illustrates a plot of log-scaled *P* values for the dummy-coded variables indicating the highest quartiles of the diet indices and the trend variables in the tests for trend. This plot is labeled to ease the identification of significant results. There were 7 cytokines with significant associations involving the AHEI-2010, 7 cytokines with associations involving the DASH index, and significant associations involving the low carbohydrate and other low carbohydrate indices. Table 3 provides the results with effect estimates from the proportional-odds models. In Table 4 are the results from logistic regression models evaluating the relationship between the *a priori* indices and TNF-α and IL-17. Higher adherence to, i.e., a higher score on, the AHEI-2010 was significantly associated with higher odds of lower IFN-γ, IL-6, IL-10, IL-17, IL-8, TNF-α, and GRO-β. The effect sizes varied. The largest effect size corresponded to IFN-γ, where, relative to the first quartile, the highest quartile of the diet index had 6.23-fold greater odds of having lower IFN-γ values. One standard deviation (Table 1) increase in the AHEI-2010 score was associated with 1.60-fold greater odds of having lower IFN-γ. The smallest estimate in the series of proportional-odds models fit with significant results was for the model with IL-10 as the response variable. The highest quartile had 3.17-fold greater odds of having lower IL-10 values relative to the first quartile and a standard deviation increase in adherence to the AHEI-2010 was associated with 1.40-fold greater odds of having lower values of IL-10, although this estimate was not significant at the α = 0.05 level. Notably, among models with AHEI-2010 as the explanatory variable, the trend tests or models specified with the scaled continuous variables were significant for all the cytokines in the table besides VEGF, TGF-∖β, and HGF. For the 2 logistic regression models, we found that the highest quartile of the AHEI-2010 was associated with 79% reduced odds of higher TNF-α values and 84% reduced odds of higher IL-17 values (Table 4). Finally, the highest quartile of adherence to the AHEI-2010 had 4.90-fold greater odds of having a lower score in the cytokine summary composite measure, compared to the first quartile, and a standard deviation increase in the score was associated with 1.55-fold greater odds of a lower cytokine summary composite score (Table 3).

**TABLE 3:** Cumulative odds ratios from proportional-odds models evaluating the relationships between the diet quality indices and select serum inflammatory cytokine values^1^

Cytokine	Q1	Q2	Q3	Q4	*P*_trend_	OR_continuous_^2^	*P*_*nonlinear*_^3^
AHEI-2010							
IFN-γ	1.00	3.52 (1.26, 9.86)∗	3.50 (1.27, 9.64)∗	6.23 (2.14, 18.16)∗∗	< 0.01∗∗	1.60 (1.11, 2.31)∗	0.08
IL-6	1.00	2.03 (0.74, 5.55)	2.16 (0.80, 5.83)	3.80 (1.34, 10.77)∗	0.02∗	1.63 (1.12, 2.37)∗	0.42
IL-10	1.00	1.67 (0.63, 4.41)	1.92 (0.74, 4.96)	3.17 (1.17, 8.58)∗	0.03∗	1.40 (0.99, 1.99)	0.11
IL-8	1.00	3.12 (1.19, 8.15)∗	2.99 (1.15, 7.73)∗	4.60 (1.68, 12.57)∗∗	< 0.01∗∗	1.32 (0.94, 1.86)	0.09
TGF-β	1.00	0.47 (0.19, 1.21)	0.98 (0.39, 2.44)	0.73 (0.28, 1.89)	0.94	0.96 (0.69, 1.35)	0.47
GRO-β	1.00	2.55 (1.00, 6.47)∗	1.86 (0.74, 4.64)	3.24 (1.24, 8.48)∗	0.04∗	1.35 (0.96, 1.89)	0.23
VEGF	1.00	1.96 (0.77, 5.00)	1.38 (0.55, 3.46)	2.02 (0.77, 5.31)	0.28	1.17 (0.84, 1.65)	0.25
HGF	1.00	1.90 (0.76, 4.77)	1.82 (0.74, 4.50)	2.05 (0.80, 5.30)	0.16	1.29 (0.92, 1.81)	0.09
Cytokine Composite^4^	1.00	2.93 (1.14, 7.57)∗	2.99 (1.18, 7.61)∗	4.90 (1.82, 13.17)∗∗	< 0.01∗∗	1.55 (1.10, 2.20)∗	0.03∗
aMED							
IFN-γ	1.00	1.27 (0.49, 3.31)	1.97 (0.75, 5.19)	1.56 (0.59, 4.10)	0.27	1.34 (0.95, 1.90)	0.13
IL-6	1.00	1.16 (0.44, 3.07)	1.18 (0.44, 3.16)	2.27 (0.85, 6.03)	0.11	1.35 (0.95, 1.93)	0.77
IL-10	1.00	1.48 (0.57, 3.83)	2.00 (0.77, 5.20)	1.55 (0.60, 4.02)	0.31	1.33 (0.95, 1.87)	0.49
IL-8	1.00	1.40 (0.56, 3.50)	1.32 (0.52, 3.37)	1.98 (0.78, 5.06)	0.18	1.19 (0.85, 1.66)	0.76
TGF-β	1.00	0.65 (0.26, 1.64)	0.69 (0.27, 1.78)	0.59 (0.23, 1.50)	0.31	0.79 (0.56, 1.10)	0.68
GRO-β	1.00	0.91 (0.37, 2.26)	1.39 (0.56, 3.48)	1.08 (0.43, 2.70)	0.71	1.14 (0.82, 1.58)	0.29
VEGF	1.00	1.72 (0.67, 4.39)	1.98 (0.77, 5.06)	1.57 (0.62, 4.01)	0.31	1.21 (0.86, 1.69)	0.87
HGF	1.00	1.23 (0.50, 3.02)	1.71 (0.68, 4.28)	0.87 (0.35, 2.16)	0.99	1.01 (0.73, 1.41)	0.08
Cytokine Composite^4^	1.00	1.77 (0.70, 4.47)	2.17 (0.86, 5.51)	1.76 (0.70, 4.45)	0.20	1.28 (0.92, 1.79)	0.93
DASH							
IFN-γ	1.00	1.16 (0.44, 3.06)	1.75 (0.66, 4.66)	2.89 (1.04, 8.01)∗	0.03∗	1.48 (1.02, 2.15)∗	0.53
IL-6	1.00	1.74 (0.64, 4.73)	2.40 (0.88, 6.54)	3.17 (1.12, 9.03)∗	0.03∗	1.63 (1.11, 2.41)∗	0.61
IL-10	1.00	1.96 (0.74, 5.24)	3.07 (1.13, 8.33)∗	4.12 (1.46, 11.58)∗∗	< 0.01∗∗	1.60 (1.10, 2.32)∗	0.96
IL-8	1.00	0.76 (0.30, 1.93)	2.61 (1.01, 6.74)∗	2.25 (0.84, 6.02)	0.04∗	1.27 (0.89, 1.81)	0.21
TGF-β	1.00	0.79 (0.32, 1.96)	0.96 (0.38, 2.42)	1.09 (0.42, 2.84)	0.78	1.15 (0.81, 1.64)	0.29
GRO-β	1.00	1.80 (0.72, 4.53)	2.50 (0.98, 6.38)	3.67 (1.38, 9.78)∗∗	< 0.01∗∗	1.50 (1.05, 2.14)∗	0.84
VEGF	1.00	1.30 (0.51, 3.33)	3.12 (1.19, 8.17)∗	2.09 (0.78, 5.62)	0.08	1.38 (0.97, 1.98)	0.12
HGF	1.00	1.84 (0.74, 4.60)	2.49 (0.98, 6.30)	1.89 (0.73, 4.95)	0.16	1.27 (0.90, 1.80)	0.12
Cytokine Composite^4^	1.00	2.05 (0.80, 5.26)	4.74 (1.79, 12.52)∗∗	3.81 (1.41, 10.31)∗∗	< 0.01∗∗	1.65 (1.15, 2.36)∗∗	0.25
Low Carbohydrate (LC)							
IFN-γ	1.00	1.10 (0.43, 2.80)	0.77 (0.30, 1.97)	1.67 (0.66, 4.24)	0.34	1.17 (0.83, 1.63)	0.76
IL-6	1.00	1.41 (0.54, 3.69)	1.06 (0.40, 2.76)	2.59 (0.99, 6.77)	0.07	1.40 (0.99, 1.98)	0.56
IL-10	1.00	1.73 (0.69, 4.39)	0.72 (0.28, 1.86)	1.78 (0.71, 4.47)	0.46	1.14 (0.82, 1.58)	0.94
IL-8	1.00	1.77 (0.71, 4.40)	0.91 (0.37, 2.25)	2.25 (0.91, 5.56)	0.18	1.31 (0.95, 1.81)	0.84
TGF-β	1.00	0.72 (0.29, 1.81)	0.49 (0.20, 1.22)	0.53 (0.21, 1.32)	0.14	0.81 (0.58, 1.12)	0.16
GRO-β	1.00	1.71 (0.70, 4.21)	0.67 (0.27, 1.64)	2.00 (0.81, 4.91)	0.33	1.18 (0.86, 1.62)	0.97
VEGF	1.00	0.85 (0.34, 2.11)	0.45 (0.18, 1.13)	1.79 (0.71, 4.48)	0.33	1.08 (0.78, 1.49)	0.45
HGF	1.00	2.11 (0.85, 5.22)	1.03 (0.42, 2.51)	2.20 (0.89, 5.40)	0.22	1.17 (0.85, 1.61)	0.47
Cytokine Composite^4^	1.00	1.28 (0.52, 3.14)	0.81 (0.33, 1.98)	1.89 (0.77, 4.65)	0.25	1.11 (0.81, 1.53)	0.79
Animal-Based LC							
IFN-γ	1.00	1.15 (0.45, 2.97)	0.67 (0.26, 1.72)	0.88 (0.33, 2.32)	0.58	0.98 (0.70, 1.37)	0.78
IL-6	1.00	1.43 (0.54, 3.75)	0.49 (0.18, 1.30)	1.64 (0.62, 4.39)	0.68	1.11 (0.79, 1.56)	0.19
IL-10	1.00	1.12 (0.44, 2.85)	0.55 (0.22, 1.41)	0.96 (0.37, 2.47)	0.64	1.03 (0.74, 1.44)	0.56
IL-8	1.00	1.16 (0.46, 2.91)	1.21 (0.49, 3.01)	2.73 (1.06, 6.99)∗	0.04∗	1.32 (0.95, 1.84)	0.86
TGF-β	1.00	0.63 (0.25, 1.60)	0.83 (0.34, 2.04)	0.43 (0.17, 1.10)	0.13	0.74 (0.53, 1.03)	0.39
GRO-β	1.00	1.28 (0.51, 3.18)	0.50 (0.20, 1.23)	1.16 (0.46, 2.91)	0.82	1.02 (0.74, 1.41)	0.46
VEGF	1.00	0.62 (0.25, 1.57)	0.48 (0.19, 1.20)	1.45 (0.56, 3.76)	0.49	1.06 (0.76, 1.48)	0.08
HGF	1.00	2.05 (0.82, 5.13)	0.91 (0.37, 2.24)	2.65 (1.04, 6.78)∗	0.14	1.29 (0.93, 1.79)	0.64
Cytokine Composite^4^	1.00	0.66 (0.26, 1.64)	0.48 (0.19, 1.19)	1.05 (0.42, 2.67)	0.97	1.01 (0.73, 1.40)	0.46
Plant-Based LC^5^							
IFN-γ	1.00	0.34 (0.09, 1.26)	3.04 (0.95, 9.74)	1.84 (0.58, 5.85)	0.07	1.44 (0.94, 2.20)	0.12
IL-6	1.00	1.15 (0.33, 3.97)	1.79 (0.54, 5.95)	2.81 (0.82, 9.66)	0.08	1.86 (1.15, 3.00)∗	0.97
IL-10	1.00	0.52 (0.16, 1.75)	1.97 (0.64, 6.08)	1.29 (0.41, 4.08)	0.34	1.27 (0.84, 1.91)	0.97
IL-8	1.00	0.88 (0.29, 2.66)	0.80 (0.27, 2.35)	0.81 (0.28, 2.38)	0.68	1.08 (0.74, 1.59)	0.94
TGF-β	1.00	0.57 (0.18, 1.80)	0.44 (0.14, 1.35)	0.47 (0.15, 1.47)	0.17	0.73 (0.48, 1.09)	0.05∗
GRO-β	1.00	0.47 (0.15, 1.46)	1.64 (0.55, 4.86)	1.31 (0.44, 3.89)	0.32	1.25 (0.84, 1.84)	0.17
VEGF	1.00	1.08 (0.36, 3.25)	1.14 (0.39, 3.31)	1.10 (0.38, 3.22)	0.84	1.02 (0.70, 1.49)	0.84
HGF	1.00	0.92 (0.31, 2.74)	1.25 (0.43, 3.64)	1.01 (0.35, 2.92)	0.89	1.15 (0.78, 1.68)	0.71
Cytokine Composite^4^	1.00	0.58 (0.19, 1.80)	2.50 (0.83, 7.50)	1.20 (0.40, 3.58)	0.38	1.27 (0.86, 1.87)	0.93

AHEI, Alternative Healthy Eating Index; aMED, Alternate Mediterranean Diet; DASH, Dietary Approaches to Stop Hypertension; GRO, growth-related oncogene; HGF, hepatocyte growth factor; IFN, interferon; LC, low carbohydrate; TGF, transforming growth factor; VEGF, vascular endothelial growth factor.

1 All models adjusted for age, sex, body mass index, tumor site, stage, human papillomavirus status, estimated caloric intake, smoking status, and drinking status. Data presented as odds ratio (95% confidence interval). *n* = 145 head and neck squamous cell carcinoma survivors. ∗∗*P* < 0.01; ∗*P* < 0.05.

2 Odds ratio (OR) corresponding to a standard deviation increase in the diet pattern score.

3 Likelihood ratio test *p*-value for a natural cubic spline model compared to specifying the model with the scaled dietary exposure.

4 CytokineSummaryScore=Z(IL6)+Z(IL8)+Z(IL17)+Z(GROβ)+Z(HGF)+Z(IFNγ)+Z(TNFα)+Z(VEGF).

5 *n* = 104 subjects in models with the plant-based low-carbohydrate index.

**TABLE 4:** Odds ratios from logistic regression models evaluating the relationships between the diet quality indices and select serum proinflammatory cytokine values (TNF-α and IL-17)^1^

Cytokine	Q1	Q2	Q3	Q4	*P*_trend_	OR_continuous_^2^	*P*_*nonlinear*_^3^
AHEI-2010							
TNF-α	1.00	0.39 (0.13, 1.14)	0.32 (0.10, 0.95)∗	0.21 (0.06, 0.66)∗∗	0.01∗	0.60 (0.38, 0.91)∗	0.35
IL-17	1.00	0.21 (0.05, 0.72)∗	0.19 (0.05, 0.70)∗	0.16 (0.04, 0.61)∗	< 0.01∗∗	0.55 (0.32, 0.91)∗	0.51
aMED							
TNF-α	1.00	0.84 (0.29, 2.42)	0.44 (0.14, 1.34)	0.40 (0.13, 1.20)	0.07	0.68 (0.45, 1.01)	0.28
IL-17	1.00	0.46 (0.12, 1.61)	0.62 (0.17, 2.11)	0.25 (0.06, 0.93)∗	0.07	0.67 (0.41, 1.05)	0.59
DASH							
TNF-α	1.00	0.58 (0.19, 1.70)	0.26 (0.08, 0.82)∗	0.31 (0.09, 0.98)∗	0.03∗	0.61 (0.39, 0.93)∗	0.61
IL-17	1.00	0.42 (0.11, 1.41)	0.25 (0.06, 0.90)∗	0.24 (0.06, 0.87)∗	0.03∗	0.57 (0.34, 0.92)∗	0.40
Low Carbohydrate							
TNF-α	1.00	0.35 (0.11, 1.05)	1.30 (0.46, 3.68)	0.18 (0.05, 0.60)∗∗	0.05∗	0.69 (0.46, 1.02)	0.82
IL-17	1.00	0.43 (0.11, 1.51)	1.57 (0.49, 5.24)	0.44 (0.11, 1.61)	0.54	0.84 (0.54, 1.30)	0.77
Animal-Based LC							
TNF-α	1.00	0.65 (0.21, 1.95)	2.13 (0.76, 6.17)	0.33 (0.09, 1.09)	0.31	0.82 (0.55, 1.21)	0.37
IL-17	1.00	0.34 (0.07, 1.34)	2.54 (0.82, 8.46)	0.53 (0.12, 2.13)	0.85	1.06 (0.68, 1.66)	0.37
Plant-Based LC^4^							
TNF-α	1.00	1.04 (0.27, 4.09)	0.46 (0.12, 1.71)	0.55 (0.15, 1.97)	0.25	0.61 (0.36, 0.98)∗	0.55
IL-17	1.00	1.82 (0.47, 7.37)	0.30 (0.05, 1.45)	0.68 (0.15, 2.92)	0.33	0.62 (0.35, 1.07)	0.64

AHEI, Alternative Healthy Eating Index; aMED, Alternate Mediterranean Diet; DASH, Dietary Approaches to Stop Hypertension; LC, low carbohydrate.

1 All models adjusted for age, sex, body mass index, tumor site, stage, human papillomavirus status, estimated caloric intake, smoking status, and drinking status. Data presented as odds ratio (95% confidence interval). *n* = 145 head and neck squamous cell carcinoma survivors. ∗∗*P* < 0.01; ∗*P* < 0.05.

2 Odds ratio corresponding to a standard deviation increase in the diet pattern score.

3 Likelihood ratio test *p*-value for a natural cubic spline model compared to specifying the model with the scaled dietary exposure.

4 *n* = 104 subjects in models with the plant-based low-carbohydrate index.

Similar, albeit marginally weaker, results were observed for models with the DASH diet quality index. The highest quartile of DASH adherence had 4.12-fold greater odds of lower IL-10 values than the first. A standard deviation increase in the DASH diet was associated with 1.60-fold greater odds of having lower IL-10 values. The response variables of the remaining models with significant associations for the DASH index included IFN-γ, IL-6, IL-8, GRO-β, TNF-α, and IL-17. Of notable interest, higher adherence to the DASH diet was significantly associated with greater odds of having a lower score on the cytokine summary composite measure. Finally, those in the highest quartile of adherence for the animal-based low-carbohydrate index had significantly higher odds of having lower IL-8 and HGF, while those in the highest quartile of the low-carbohydrate index had 82% reduced odds of having high TNF-α values.

## Discussion

We present results highlighting the relevance of a priori-defined diet quality indices in influencing serum proinflammatory cytokine levels in a cohort of newly diagnosed HNSCC patients. Our analysis found significant inverse associations between the AHEI-2010 diet quality index and levels of a host of proinflammatory cytokines. To a lesser extent, we observed some inverse associations involving the DASH diet index.

In a previous analysis involving the same patient cohort, we reported strong inverse associations between adherence to the AHEI-2010 and the risks of all-cause and cancer-specific mortality [16]. We evaluated the same diet quality indices employed in this study and found that only the AHEI-2010 was consistently and inversely associated with mortality. The findings from this analysis corroborate those results above and suggest a biologically plausible mechanism for explaining the bolstered survival profiles of those most adherent to the AHEI-2010 within this cohort. The results we observed were robust across several model specifications and cytokines. Furthermore, we computed a proinflammatory cytokine summary composite metric to gauge the overall inflammatory burden and found a significant inverse association with the AHEI-2010 and DASH scores.

The AHEI was developed in 2002 and updated in 2010 as an alternative to the Healthy Eating Index and emphasized foods and nutrients related to chronic illness incidence [23]. Since its inception, adherence to the AHEI-2010 has strongly predicted chronic disease in several large epidemiological studies [24,[31], [32], [33], [34], [35], [36], [37], [38], [39]]. Additionally, there have been some reports in the literature relating adherence to this diet index and markers of inflammation. Most notably, Fung et al. [24] reported inverse associations between higher adherence of consumption to the AHEI and levels of serum C-reactive protein (CRP), a global measure of systemic inflammation, and IL-6. In a more recent analysis involving the Melbourne Collaborative Cohort study, Li et al. [40] reported significant inverse associations between AHEI-2010 adherence and IL-6, CRP, and IFN-γ levels in a cohort of Australian participants of primarily White-European descent. Similarly, other studies examining the relationship between AHEI-2010 and systemic markers of inflammation have reported inverse associations between adherence to this index and several biomarkers [34,[41], [42], [43]]. The results we present join this bounty of evidence in highlighting a potential beneficial impact of AHEI-2010 adherence on systemic markers of inflammation. Nevertheless, our analysis involved a more comprehensive panel of proinflammatory cytokines and is the first to report these relationships in the HNSCC population.

In addition to the AHEI-2010, the DASH index was associated with lower levels of several proinflammatory cytokines. However, in the cited survival analysis we describe above, no significant or robust associations were reported between DASH index adherence throughout the first 3 y of follow-up and survival in this cohort [16], although the relationships we observed between the DASH index and the proinflammatory cytokines in this analysis were not as pronounced as those seen in the AHEI-2010 index. Nonetheless, we must qualify these results in light of those observed for the AHEI-2010 and posit that other components of the AHEI-2010 may procure better survival beyond just the potential effects on systemic inflammation.

The relationship between inflammation and cancer is not a novel one. Inflammation—local, organ-specific, or systemic—is understood to influence the tumor microenvironment and alter tumor growth dynamics [44]. An abundance of proinflammatory cytokines and other chemokines that is not checked by the presence of a commensurate quantity of anti-inflammatory cytokines and chemokines may result in neovascularization through angiogenesis and rapid tumor growth [45]. At the cellular level, oxidative insults generate an inflammatory cascade that upregulates critical transcription factors in cancer cells, such as HIF1-α, STAT3, and NF-κB. In turn, these transcription factors, particularly NF-κB, upregulate the transcription of proinflammatory cytokines [46]. A positive feedback loop ensues whereby these cytokines, such as IL-6, IL-8, and TNF-α, amplify the inflammatory infiltrate in the tumor microenvironment and systemically [[46], [47], [48], [49]]. In particular, TNF-α and IL-6 have garnered considerable attention in the context of cancer, being labeled master regulators of tumor-related inflammation [48]. Consequently, the transcription factors activated and noted above bind to consensus sequences in the promoter regions of genes crucial to transformation and proliferation, such as metalloproteinases, COX-2, cyclin D1, and several other crucial players [46,[50], [51], [52]]. Thus, targets in the pathways discussed and inflammation-dampening have been implicated as attractive therapeutic modalities in cancer research [[53], [54], [55], [56]]. Signaling through these pathways is also subject to modulation by nutrients, which can subsequently abate the expression of several proinflammatory cytokines. In particular, polyphenols have received a great deal of scrutiny for their suppressive actions on players in these pathways [[57], [58], [59]]. Thus, these previous findings, albeit mainly derived from animal and basic science research, support the biological plausibility of the results of our study.

The study has several strengths, including the use of objective laboratory markers of inflammation, a comprehensive and validated FFQ for ascertaining dietary intake, and adjustment for multiple known confounders. Despite the strengths of the analysis, there are limitations we must highlight. Primarily, the study’s cross-sectional design precludes any causal inference from being made, and reverse causality, specifically, cannot be ruled out. Furthermore, we had a limited sample size of subjects from the larger pool of UM-SPORE participants with laboratory data, which may have resulted in reduced statistical power, and we were not able to further adjust for other covariates. Additionally, the sample was comprised primarily of non-Hispanic White subjects, which limits the generalizability of the results. Nevertheless, we were able to identify cogent associations that were supported by some of our previous findings from this cohort study and the work of others. Despite several strengths in implementing a validated FFQ for capturing usual food intake, we must qualify that systematic measurement errors may occur in specific contexts. Finally, as with any observational study design, residual confounding by other unmeasured characteristics or clinical features cannot be ruled out.

In summary, pretreatment adherence to the AHEI-2010 and DASH indices may result in a more favorable profile of serum proinflammatory cytokines in newly diagnosed HNSCC patients. In particular, the AHEI-2010 appears to be a robust dietary indicator in HNSCC when considering our results in light of previous findings suggesting improved survival in this cohort when adhering more closely to those guidelines. Nonetheless, we clarify that using only pretreatment dietary intake values and the cross-sectional design limit causal inference. Given our findings, replicating these results in other cohort studies and implementing interventional or other study designs investigating the relationships between these diet quality indices and serum proinflammatory cytokine levels in newly diagnosed HNSCC patients is warranted.

## Author contributions

The authors’ responsibilities were as follows—CAMV, LSR, GTW: designed research (project conception, development of overall research plan, and study oversight); CAMV: analyzed data and performed statistical analysis; CAMV, AEA: wrote paper; CAMV: had primary responsibility for final content; and all authors: read and approved the final manuscript.

## Funding

This research was supported by NIH/NCI P50CA097248 and USDA-NIFA Hatch Project 1011487. CAMV was supported by a fellowship from the Robert Wood Johnson Health Policy Research Scholars Program, a national leadership program supported by the Robert Wood Johnson Foundation. It supports scholars from diverse disciplines and backgrounds in applying and advocating for policy change that improves health and equity. The project described in this article is supported by the program. The views expressed here do not necessarily reflect the views of the Foundation. www.healthpolicyresearch-scholars.org. The funders had no role in the design of the study; in the collection, analyses, or interpretation of data; in the writing of the manuscript, or in the decision to publish the results.

## Data availability

Data described in the manuscript, code book, and analytic code will be made available upon request pending application and approval. The R code for reproducing the analyses is publicly available and found in a GitHub repository [30].

## Conflict of interest

The authors report no conflicts of interest.

## Appendix A Supplementary data

Multimedia component1 Multimedia component1
