# Supplementary material for: Associations Between Diet Quality and Proinflammatory Cytokines in Newly Diagnosed Head and Neck Cancer Survivors
Source: Curr Dev Nutr. 2023 Oct 12;7(11):102015. doi: 10.1016/j.cdnut.2023.102015 (PMC10641111; doi:10.1016/j.cdnut.2023.102015)
Supplement: Multimedia component1 [file mmc1.docx]

**
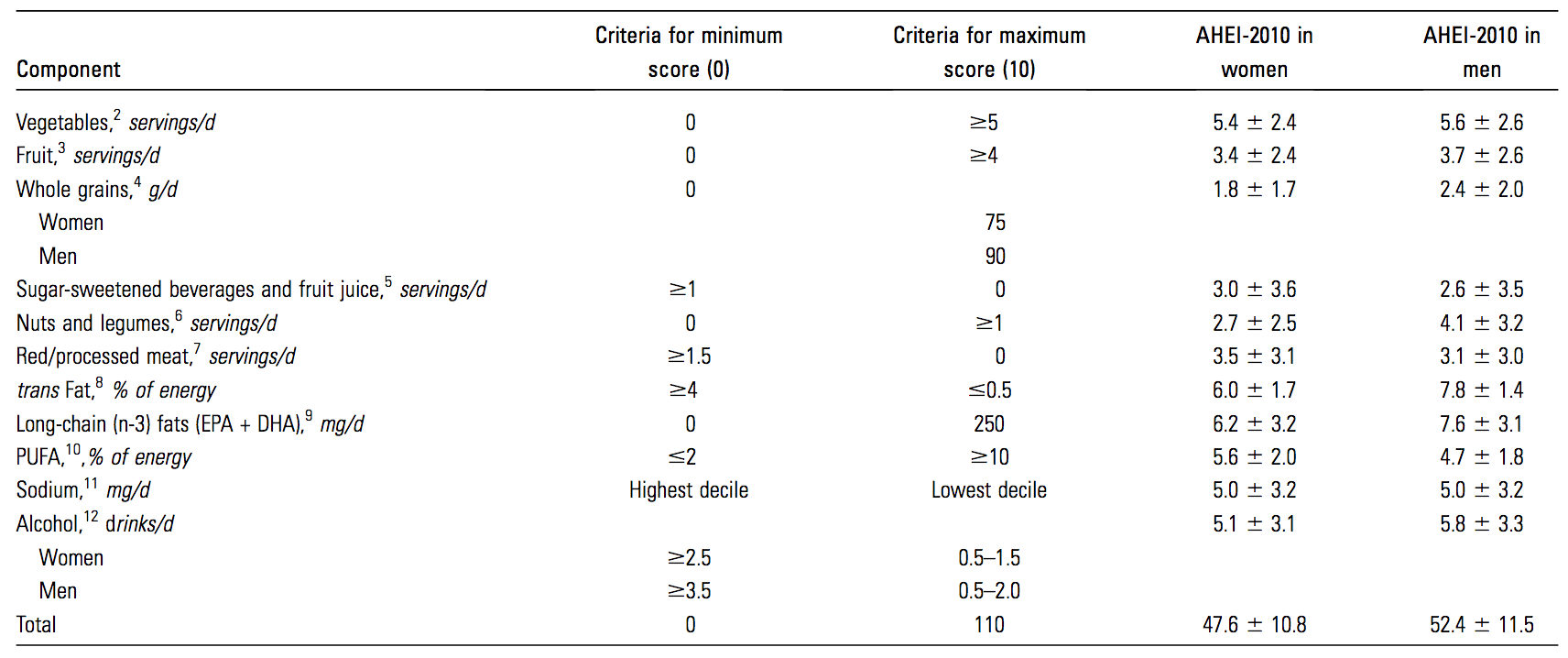
Supplementary Tables and Figures**

**Supplementary Figure 1**. Scoring algorithm for the AHEI-2010 index. This table is taken from the original publication (we cite it here) and is not the intellectual property of the authors of this publication (1).


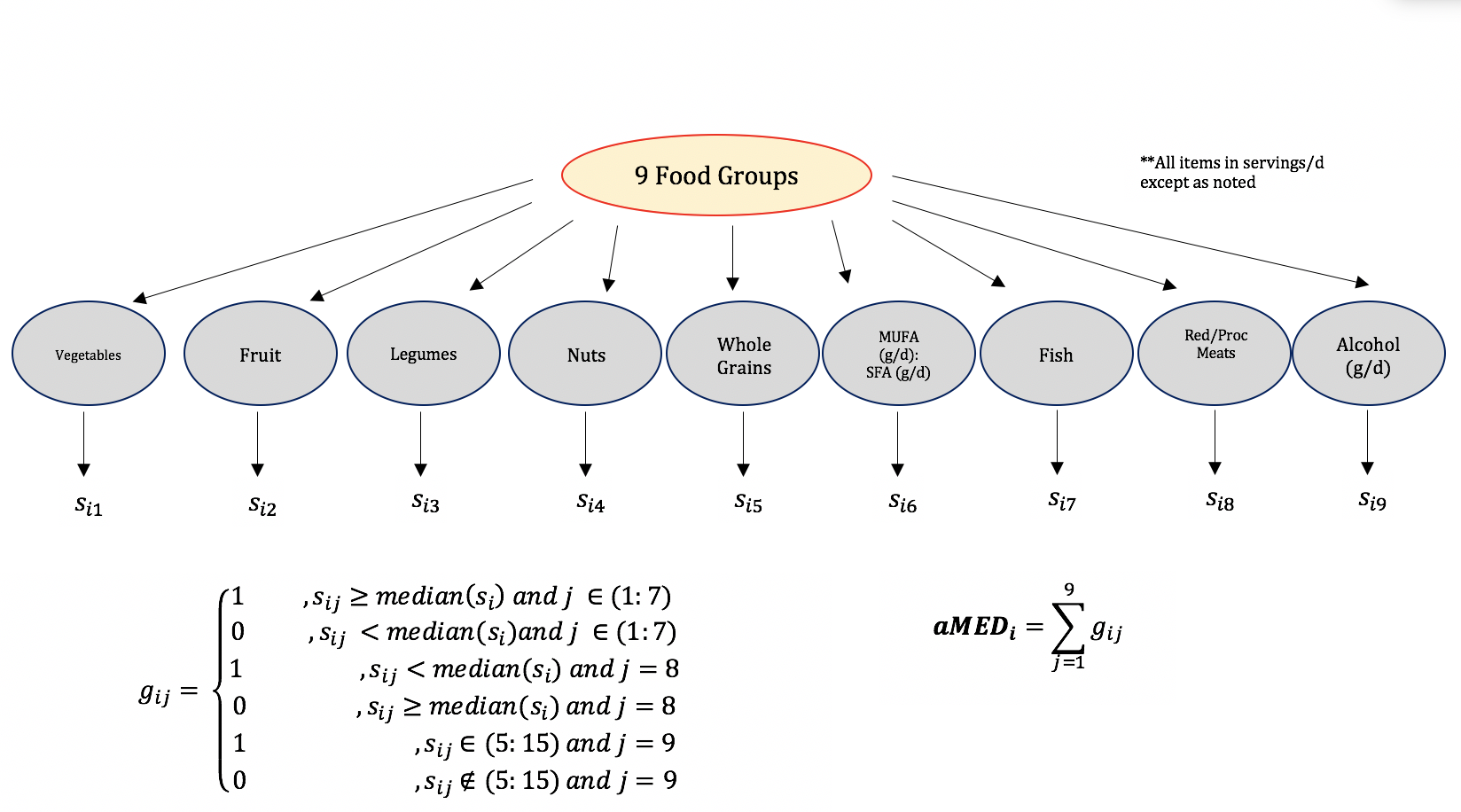


**Supplementary Figure 2**. Scoring algorithm for the aMED index.


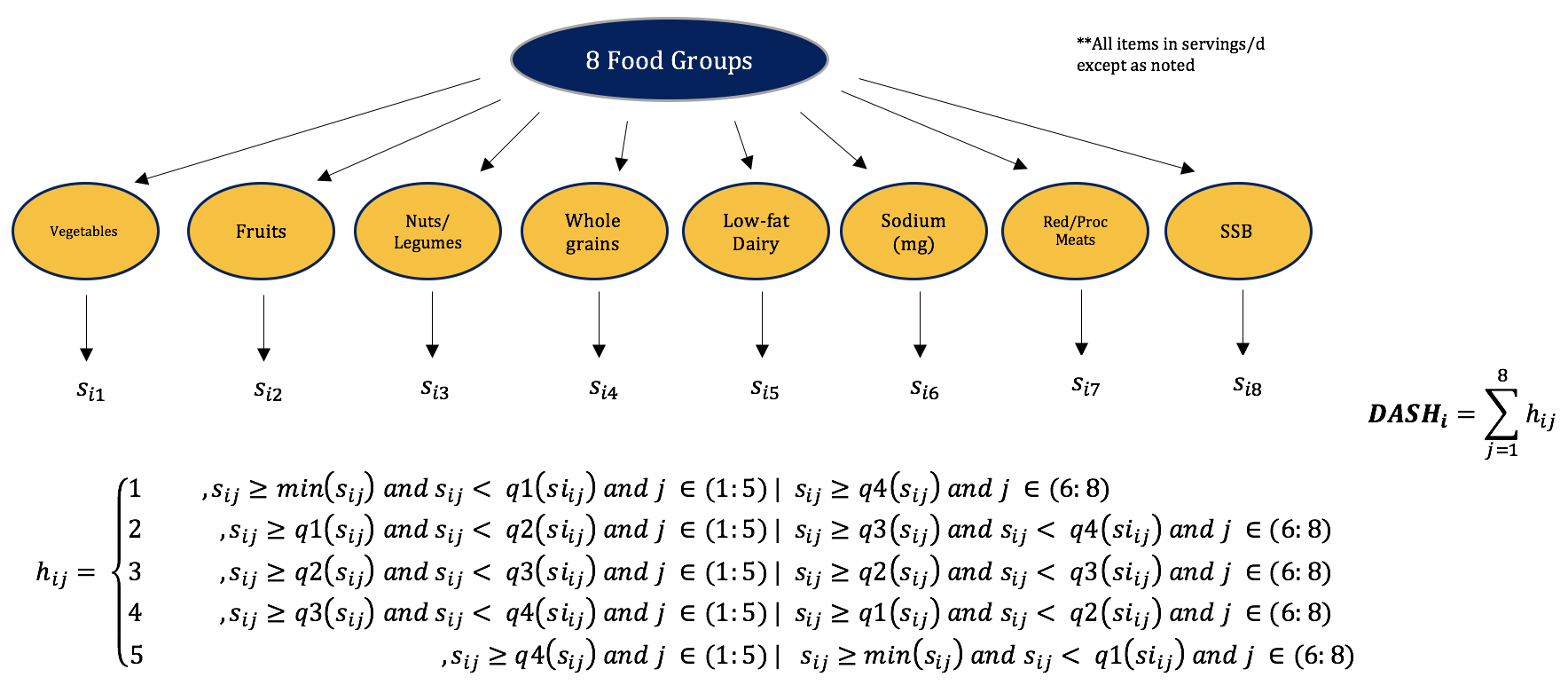


**Supplementary Figure 3**. Scoring algorithm for the DASH index.


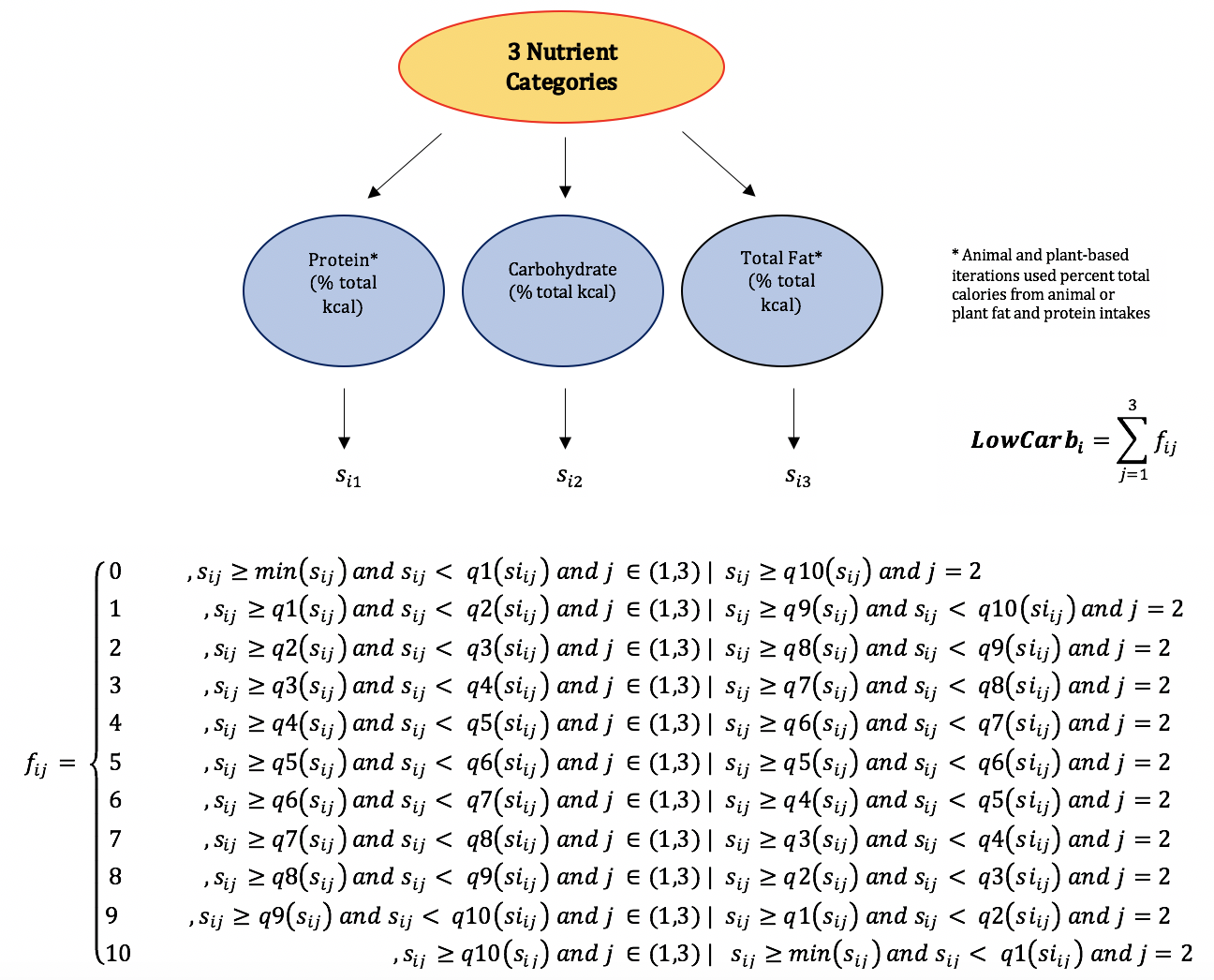


**Supplementary Figure 4**. Scoring algorithm for the low carbohydrate indices.


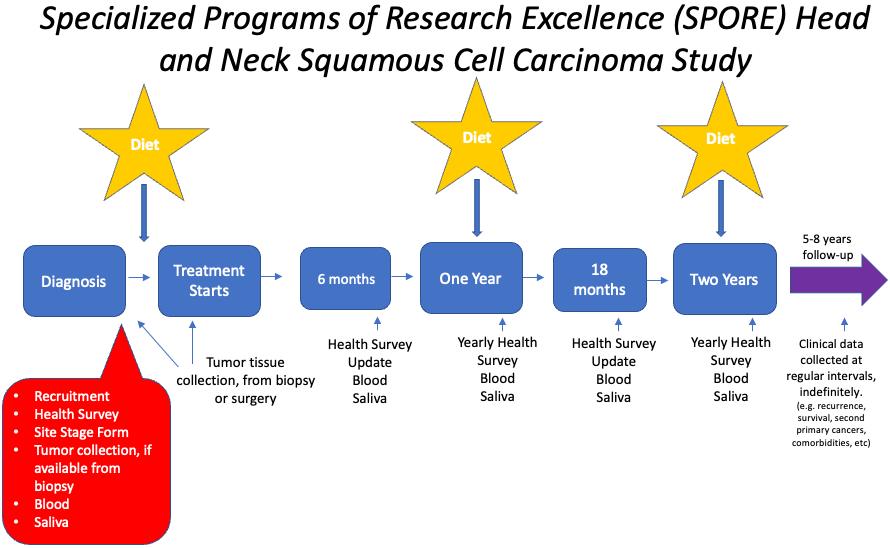


**Supplementary Figure 5**. Study design and trajectory of the SPORE study.

“Pretreatment”

# References

1. Chiuve SE, Fung TT, Rimm EB, Hu FB, McCullough ML, Wang M, et al. Alternative dietary indices both strongly predict risk of chronic disease. J Nutr. 2012 Jun;142(6):1009–18.
